# Discomfort Experienced Due to the Odor and Physiological Responses of Residual Tobacco Smoke Brought Into Workplaces by Smokers on Work Performance and Mental Health

**DOI:** 10.2188/jea.JE20240354

**Published:** 2025-09-05

**Authors:** Kosuke Kiyohara, Takaaki Ikeda, Tomohiro Ishimaru, Ryo Okubo, Takahiro Tabuchi

**Affiliations:** 1 Department of Food Science, Faculty of Home Economics, Otsuma Women’s University, Tokyo, Japan; 2 Department of Health Policy Science, Graduate School of Medical Science, Yamagata University, Yamagata, Japan; 3 Department of Medical Humanities, School of Medicine, University of Occupational and Environmental Health, Japan, Kitakyushu, Japan; 4 Department of Clinical Epidemiology, Translational Medical Center, National Center of Neurology and Psychiatry, Tokyo, Japan; 5 Cancer Control Center, Osaka International Cancer Institute, Osaka, Japan; 6 Division of Epidemiology, School of Public Health, Tohoku University Graduate School of Medicine, Miyagi, Japan

**Keywords:** residual tobacco smoke, presenteeism, mental health, smoke-free policy, thirdhand smoke

## Abstract

**Background:**

The discomfort experienced due to residual tobacco smoke, a form of thirdhand smoke exposure brought into workplaces by smokers, and its health impacts on non-smokers have been inadequately investigated. This study explored associations between non-smokers’ discomfort and work performance and mental health.

**Methods:**

This observational internet-based survey was conducted in 2021 as part of the Japan Society and New Tobacco Internet Survey. Participants comprised 6,519 adult workers without firsthand or secondhand smoking. Work performance and mental health were evaluated using the Work Functioning Impairment Scale (WFun) and Kessler Psychological Distress Scale (K6), respectively. The proportion of participants who experienced discomfort from the residual tobacco smoke in their workplace by smokers in the previous year was calculated according to the workplace’s smoke-free policy, and the difference was assessed using the χ^2^ test. The association between such discomfort and WFun and K6 scores was examined using univariable and multivariable logistic regression analyses.

**Results:**

Among respondents, 17.1% reported experiencing discomfort due to the residual tobacco smoke. A strict smoke-free workplace policy was associated with a lower proportion of respondents experiencing such discomfort (*P* < 0.001). Those who experienced discomfort more frequently had significantly higher scores on the WFun (15.5% “never”, 21.3% “sometimes”, 26.2% “frequently”) and K6 (37.8% “never”, 48.2% “sometimes”, 50.8% “frequently”). Adjusting for potential covariates in multivariable analyses did not change these results.

**Conclusion:**

Discomfort from thirdhand smoke was associated with worse work performance and mental health problems. Promotion of strict smoke-free workplace policies is required to reduce such experiences.

## INTRODUCTION

The negative effects of smoking include firsthand smoke from the smoker’s mainstream smoke, which contains harmful components, and secondhand smoke from side-stream smoke generated during smoking and from exhaled smoke.^1^ The potential exposure to residual tobacco smoke, which is tobacco smoke residue from cigarettes and other tobacco products, known as thirdhand smoke, has also been recently discussed.^2^^–^^4^ Even when the tobacco smoke is not visible, individuals may be exposed to residual tobacco smoke in their daily lives.

Residual tobacco smoke contains chemicals, such as nicotine, 3-ethenyl pyridine, phenol, cresol, naphthalene, formaldehyde, and tobacco-specific nitrosamines, which produce foul odors^5^^–^^7^ and cause discomfort to the exposed person. Residual tobacco smoke is not only adsorbed in the walls and floors of rooms where smoking is allowed but is also brought into a non-smoking indoor environment through absorption on the smoker’s clothing or body.^8^ As residual tobacco smoke can persist in smokers’ breath and clothes for a long time after smoking,^6^ an unpleasant odor is also brought into various places as smokers move around.

According to a recent report,^3^ thirdhand smoke contains over 20 chemicals known to cause cancer or reproductive harm, and laboratory and in vivo studies have shown that thirdhand smoke exposure can lead to DNA damage, cellular stress responses, cytotoxic effects, metabolic changes, oncogenic phenotypes, increased lung cancer risk, behavioral changes, impaired wound healing, and immune system alterations. A human study also demonstrated that thirdhand smoke exposure activates stress response pathways, including DNA repair and mitochondrial activity.^9^

However, there is limited evidence on the impact of thirdhand smoke on human health in real-world settings. Although several observational studies suggest that thirdhand smoke exposure may increase risks, such as cervical cancer in nonsmoking women,^10^ affect insufficient and excess sleep duration in adolescents,^11^ and lead to gastrointestinal issues in children,^12^ little is known about the impact of discomfort caused by the odor and physiological responses of residual tobacco smoke brought into the workplace by smokers. Since malodorous compounds in residual tobacco smoke may have adverse psychological and physiological effects on the exposed person, if workers experience such discomfort in indoor working environments, their work performance and mental health may be adversely affected. Although randomized controlled trials (RCTs) are typically necessary to obtain strong empirical evidence, it is ethically impossible to conduct an RCT on such experiences of discomfort, making investigations through observational studies crucial.

The Japan Society and New Tobacco Internet Survey (JASTIS) is an Internet-based longitudinal cohort study designed to investigate the perceptions, attitudes, and use of various types of tobacco products (eg, heated tobacco products [HTPs], electronic cigarettes, and combustible tobacco) in Japan.^13^ Using data from the JASTIS questionnaire survey conducted in 2021, this study examined the experience of discomfort due to the odor and physiological responses of residual tobacco smoke brought into the workplace by smokers, targeting Japanese workers without either firsthand or secondhand smoking. We hypothesized that the frequency of such experiences is associated with smoke-free workplace rules. Furthermore, we explored the associations between these experiences, work performance, and mental health conditions.

## METHODS

### Study design of the JASTIS

The JASTIS research profile has been previously reported in detail.^13^ Launched in 2015, the JASTIS conducts an annual Internet-based self-reported questionnaire survey among survey panelists who are randomly selected and/or invited for follow-up. Each survey is closed when the target number of respondents completes the questionnaire. Respondents are recruited from a large survey panel managed by Rakuten Insight, a nationwide Internet research agency. Rakuten Insight has a pool of 2.3 million panelists covering various social categories, such as education, housing tenure, and marital status, as defined by the Japanese census.^13^ In this study, we used cross-sectional data from a survey conducted among 26,000 people in February 2021.

### Quality control

In internet-based surveys, incorrect or inconsistent responses may occur, leading to random measurement errors that can increase data variability and potentially result in misclassification.^14^^,^^15^ As in previous survey reports,^16^^–^^18^ closely related questions in the same survey were checked to ensure the consistency of responses and identify erroneous data. To reduce the potential risk of overestimation or underestimation of measures and improve data quality, invalid responses were excluded from the database.

### Study participants

Among the JASTIS survey respondents in 2021, non-smoking adult workers were included in the analysis. Respondents who were minor students, retirees, housewives, or unemployed individuals at the time of the survey and those who had not gone to the workplace in the last year were excluded. Furthermore, to avoid the effects of firsthand and secondhand smoking on the outcomes, those who had used combustible tobacco or HTPs in the previous year and those who had experienced passive smoking (exposure to environmental tobacco smoke) in the workplace were excluded.

### Data collection

The following information was obtained from the JASTIS 2021 survey: discomfort experienced due to residual tobacco smoke brought into the workplace by smokers in the previous year, smoke-free workplace policy, presenteeism, mental health condition, sex, age, prefecture, past tobacco use (combustible tobacco and HTPs), experience of passive smoking in the workplace, occupation, working hours per week, final education, number of people in the household, marital status, history of respiratory diseases (asthma, pneumonia, bronchitis, and COPD), and history of mental illness. The discomfort experienced due to residual tobacco smoke in the workplace was assessed using the following question: “In the last year, have you ever felt uncomfortable, sick, or coughed because of the breath or odor of tobacco emitted by smokers sitting next to you or around you?” Respondents were asked to answer this question regarding their workplace using the following options: “never,” “sometimes,” or “frequently.” The proportion of coronavirus disease 2019 (COVID-19) cases per population in each prefecture was calculated based on the number of COVID-19 cases reported between January 15, 2020, and February 7, 2021. Following a previous study,^19^ the respondents were divided into two groups according to whether the number of infections per population in the prefecture in which they lived was higher or lower than the average number of COVID-19 infections per population in Japan.

### Outcome measures

Work performance was measured using the Work Functioning Impairment Scale (WFun), a scale assessing presenteeism, which refers to the practice of continuing to work despite being sick or being of poor health.^20^ The WFun consists of seven items rated on a 5-point scale, with a higher total score indicating greater impairment in work functioning.^20^ Based on a previous study,^21^ a total score of 21 or higher was classified as high impairment in work functioning.

Mental health status was assessed using the Kessler Psychological Distress Scale (K6), which consists of six items rated on a 5-point scale, with a higher total score indicating worse mental health.^22^^,^^23^ A total score of 5 or higher was defined as suspicion of depression/anxiety.

### Statistical analysis

The proportion of participants who had experienced discomfort due to the smell of residual tobacco smoke brought into their workplace by smokers in the previous year was calculated according to the smoke-free policy of the workplace, and the difference was assessed using the χ^2^ test. The association between such discomfort and WFun and K6 scores was examined using univariate and multivariable logistic regression analyses to calculate odds ratios (ORs) and their associated 95% confidence intervals (CIs). Three multivariable models were evaluated to adjust for the covariates. Model 1 was adjusted for sex and age. Model 2 was adjusted for prefecture (living in a prefecture with a high or low proportion of COVID-19 cases), past use of combustible tobacco and heated tobacco products, occupational classification, working hours per week, final education, number of people in the household, and marital status in addition to the covariates included in model 1. Model 3 was adjusted for a history of respiratory diseases and mental illness in addition to the covariates included in model 2.

Furthermore, in subgroup analyses, the association between the experience of discomfort and WFun and K6 was examined according to the history of respiratory diseases and mental illness. All tests were two-tailed, and a *P*-value of <0.05 was considered statistically significant. All statistical analyses were performed using SPSS v27.0 J (IBM Corp., Armonk, NY, USA).

### Ethics

This study was approved by the Research Ethics Committee (blinded for review). The Internet panelists of Rakuten Insight had already agreed to participate in different research surveys at the time of previous surveys. They were given the option of not responding to any part of the questionnaire and discontinuing the survey at any point. They were identified using research-specific numbers, and no personal identifiers were collected for this survey.

### Patient and public involvement statement

No funding was available to support patients or members of the public in the study design, interpretation of results, or development of the dissemination strategy. We appraised the registry-based data and analyzed them without public or patient involvement.

## RESULTS

### Discomfort due to the odor and physiological responses of residual tobacco smoke brought into workplaces by smokers

**Figure 1.**
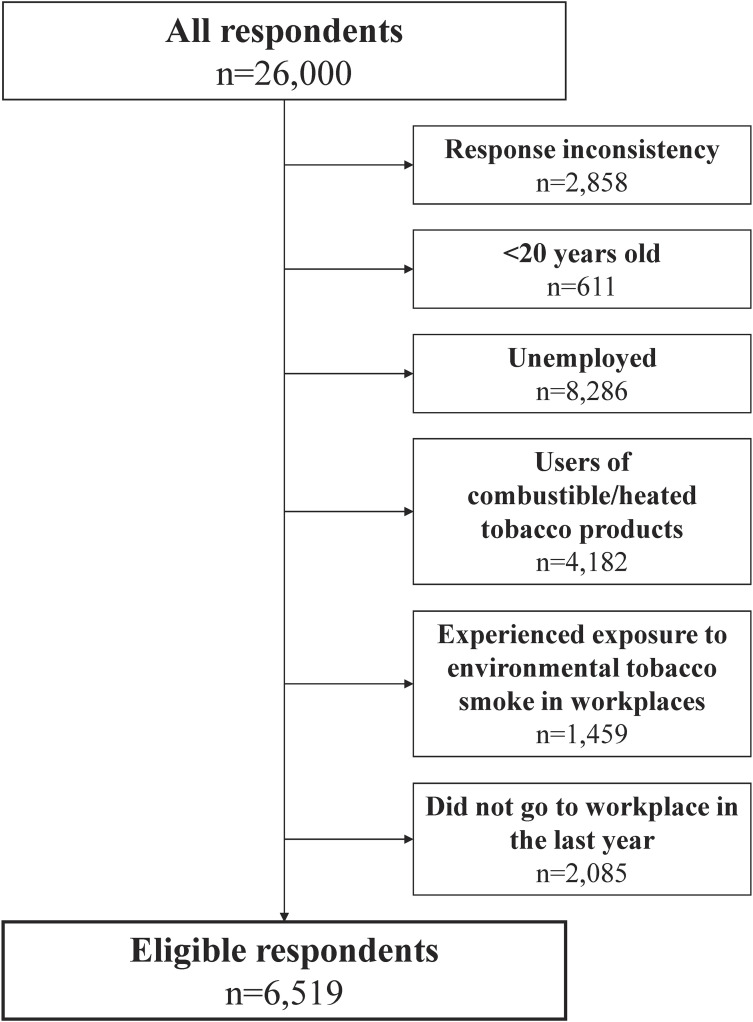
Flowchart of the selection of study participants from the JASTIS 2021 survey.

**Figure 2.**
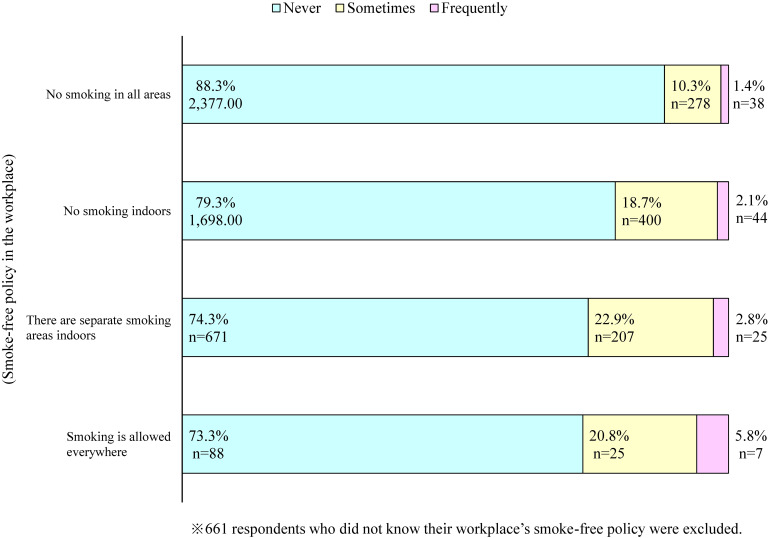
The proportion of respondents who experienced discomfort due to the odor and physiological responses of residual tobacco smoke in the workplace according to the smoke-free workplace policy.

Figure 1 shows the eligible study participants selected from the JASTIS 2021 survey. Among the 26,000 respondents, 6,519 non-smoking adult workers (male: 51.8%) were included in the current analysis. Table 1 shows the discomfort experienced due to the odor and physiological responses of residual tobacco smoke brought into workplaces by smokers in the previous year, according to the respondents’ characteristics. Overall, 17.1% of the respondents experienced such discomfort in the previous year (15.1% “sometimes” and 1.9% “frequently”). Respondents who were younger (*P* = 0.006), worked longer hours per week (*P* = 0.025), had a lower educational level (*P* = 0.027), a history of respiratory diseases (*P* = 0.002), and a history of mental illness (*P* = 0.034) were more likely to experience such discomfort. Occupational classification was also associated with discomfort (*P* = 0.022). Figure 2 shows the proportion of respondents who experienced discomfort according to the smoke-free workplace policy. A strict smoke-free workplace policy was significantly associated with a lower proportion of workers who experienced discomfort (*P* < 0.001). The proportion of those who experienced discomfort in the previous year was 11.7% in workplaces with a “No smoking in all areas” policy. In comparison, it was 26.7% in workplaces with a “Smoking is allowed everywhere” policy.

**Table 1.** Discomfort experienced due to odor and physiological responses of residual tobacco smoke brought into workplaces by smokers in the previous year according to respondents’ characteristics

		Discomfort experienced in the previous year						Total	*P*-value

		Never		Sometimes		Frequently			
		*n*	(%)	*n*	(%)	*n*	(%)	*N*	
Sex	Male	2,800	(82.8%)	511	(15.1%)	69	(2.0%)	3,380	0.804
	Female	2,607	(83.1%)	475	(15.1%)	57	(1.8%)	3,139	

Age, years	20–39	1,678	(82.1%)	324	(15.8%)	43	(2.1%)	2,045	0.006
	40–59	2,534	(82.1%)	485	(15.7%)	67	(2.2%)	3,086	
	≥60	1,195	(86.1%)	177	(12.8%)	16	(1.2%)	1,388	

Prefecture	High proportion of COVID-19 cases	3,026	(83.2%)	539	(14.8%)	73	(2.0%)	3,638	0.668
	Low proportion of COVID-19 cases	2,381	(82.6%)	447	(15.5%)	53	(1.8%)	2,881	

Combustible tobacco	Never smoked	3,820	(83.6%)	660	(14.4%)	89	(1.9%)	4,569	0.064
	Past smokers	1,587	(81.4%)	326	(16.7%)	37	(1.9%)	1,950	

Heated tobacco products	Never used	5,261	(83.1%)	947	(15.0%)	122	(1.9%)	6,330	0.095
	Past users	146	(77.2%)	39	(20.6%)	4	(2.1%)	189	

Occupational classification	Administrative and managerial workers/clerical workers	1,956	(81.9%)	385	(16.1%)	48	(2.0%)	2,389	0.022
	Professional and technical workers	1,229	(84.6%)	200	(13.8%)	23	(1.6%)	1,452	
	Sales workers/service workers/security workers	876	(80.4%)	191	(17.5%)	22	(2.0%)	1,089	
	Others	1,346	(84.7%)	210	(13.2%)	33	(2.1%)	1,589	

Working hours per week	<20	903	(86.2%)	134	(12.8%)	11	(1.0%)	1,048	0.025
	20–29	659	(83.8%)	114	(14.5%)	13	(1.7%)	786	
	30–39	1,109	(82.9%)	206	(15.4%)	22	(1.6%)	1,337	
	40–49	2,055	(81.4%)	406	(16.1%)	64	(2.5%)	2,525	
	≥50	681	(82.7%)	126	(15.3%)	16	(1.9%)	823	

Final education	Junior high school/high school	1,059	(80.0%)	232	(17.5%)	33	(2.5%)	1,324	0.027
	Junior college/technical college	1,255	(83.3%)	224	(14.9%)	27	(1.8%)	1,506	
	University	2,679	(83.5%)	474	(14.8%)	56	(1.7%)	3,209	
	Graduate school	414	(86.3%)	56	(11.7%)	10	(2.1%)	480	

Number of people in household (including the respondent)	1	1,141	(81.7%)	232	(16.6%)	24	(1.7%)	1,397	0.187
	≥2	4,266	(83.3%)	754	(14.7%)	102	(2.0%)	5,122	

Marital status	Married	3,245	(83.6%)	563	(14.5%)	72	(1.9%)	3,880	0.198
	Not married	2,162	(81.9%)	423	(16.0%)	54	(2.0%)	2,639	

History of respiratory disease	Yes	786	(79.6%)	185	(18.7%)	16	(1.6%)	987	0.002
	No	4,621	(83.5%)	801	(14.5%)	110	(2.0%)	5,532	

History of mental illness	Yes	519	(79.4%)	121	(18.5%)	14	(2.1%)	654	0.034
	No	4,888	(83.3%)	865	(14.7%)	112	(1.9%)	5,865	

Total		5,407	(82.9%)	986	(15.1%)	126	(1.9%)	6,519	

COVID-19, coronavirus disease 2019.

### Association with work performance and mental health status

Table 2 shows the association between the discomfort experienced due to the odor and physiological responses of residual tobacco smoke brought into the workplace by smokers and the WFun scores. The proportion of respondents with high WFun scores was 15.5% among those who had “never” experienced such discomfort. In comparison, it was 21.3% and 26.2% among those who had “sometimes” and “frequently” experienced it, respectively. Compared with respondents who had “never” experienced discomfort, the proportion of those with high WFun scores was significantly high among those who had “sometimes” (unadjusted OR 1.47; 95% CI, 1.24–1.74) and “frequently” (unadjusted OR 1.93; 95% CI, 1.29–2.89) experienced it. In the three multivariable logistic regression models, adjusting for potential covariates did not affect the results. For example, in the fully adjusted model (model 3), ORs for those who experienced discomfort “sometimes” (adjusted OR 1.39; 95% CI, 1.16–1.66) and “frequently” (adjusted OR 1.84; 95% CI, 1.21–2.79) were significantly high. Table 3 shows the association between the discomfort experienced due to the odor and physiological responses of residual tobacco smoke brought into the workplace by smokers and K6 scores. The proportion of respondents with high K6 scores was 37.8%, 48.2%, and 50.8% among those who had “never,” “sometimes,” and “frequently” experienced such discomfort, respectively. Compared with respondents who had “never” experienced discomfort, the proportion of those with high K6 scores was significantly high among those who had “sometimes” (unadjusted OR 1.53; 95% CI, 1.33–1.75) and “frequently” (unadjusted OR 1.70; 95% CI, 1.19–2.42) experienced it. In the three multivariable logistic regression models, adjusting for potential covariates did not affect the results. For example, in the fully adjusted model (model 3), ORs for those who experienced discomfort “sometimes” (adjusted OR 1.46; 95% CI, 1.26–1.69) and “frequently” (adjusted OR 1.64; 95% CI, 1.13–2.37) were significantly high. eTable 1 and eTable 2 show the association between the experience of discomfort and WFun and K6 according to participants’ history of respiratory diseases and mental illness. Regardless of their medical history, as in the overall analysis, workers who experienced discomfort more frequently were likely to have high scores on the WFun and K6.

**Table 2.** Association between discomfort experienced due to odor and physiological responses of residual tobacco smoke brought into workplaces by smokers and presenteeism (WFun)

			WFun score		Univariable analysis		Multivariable analysis					
	
			≥21				Model 1		Model 2		Model 3	
		*N*	*n*	(%)	OR	(95% CI)	OR	(95% CI)	OR	(95% CI)	OR	(95% CI)
Discomfort experienced due to odor of residual tobacco smoke brought into workplaces by smokers in the previous year	Never	5,407	840	(15.5%)	ref.		ref.		ref.		ref.	
	Sometimes	986	210	(21.3%)	1.47	(1.24–1.74)	1.43	(1.21–1.70)	1.44	(1.21–1.71)	1.39	(1.16–1.66)
	Frequently	126	33	(26.2%)	1.93	(1.29–2.89)	1.80	(1.19–2.70)	1.83	(1.21–2.76)	1.84	(1.21–2.79)

Sex	Male	3,380	588	(17.4%)			1.19	(1.04–1.36)	1.06	(0.91–1.23)	1.08	(0.93–1.26)
	Female	3,139	495	(15.8%)			ref.		ref.		ref.	

Age, years	20–39	2,045	442	(21.6%)			3.42	(2.72–4.29)	2.94	(2.29–3.77)	2.74	(2.13–3.53)
	40–59	3,086	537	(17.4%)			2.60	(2.08–3.24)	2.40	(1.90–3.02)	2.22	(1.76–2.81)
	≥60	1,388	104	(7.5%)			ref.		ref.		ref.	

Prefecture	High proportion of COVID-19 cases	3,638	610	(16.8%)					0.92	(0.80–1.05)	0.91	(0.80–1.05)
	Low proportion of COVID-19 cases	2,881	473	(16.4%)					ref.		ref.	

Use of combustible tobacco	Never smoked	4,569	775	(17.0%)					ref.		ref.	
	Past smokers	1,950	308	(15.8%)					1.10	(0.93–1.29)	1.03	(0.87–1.21)

Use of heated tobacco products	Never used	6,330	1,042	(16.5%)					ref.		ref.	
	Past users	189	41	(21.7%)					1.25	(0.86–1.82)	1.29	(0.88–1.90)

Occupational classification	Administrative and managerial workers/clerical workers	2,389	420	(17.6%)					ref.		ref.	
	Professional and technical workers	1,452	273	(18.8%)					0.93	(0.78–1.12)	0.94	(0.78–1.12)
	Sales workers/service workers/security workers	1,089	165	(15.2%)					0.81	(0.66–0.99)	0.80	(0.65–0.98)
	Others	1,589	225	(14.2%)					0.88	(0.73–1.07)	0.88	(0.73–1.06)

Working hours per week	<20	1,048	128	(12.2%)					ref.		ref.	
	20–29	786	86	(10.9%)					0.81	(0.60–1.09)	0.83	(0.62–1.12)
	30–39	1,337	213	(15.9%)					1.04	(0.81–1.34)	1.07	(0.83–1.38)
	40–49	2,525	459	(18.2%)					1.08	(0.86–1.36)	1.16	(0.92–1.46)
	≥50	823	197	(23.9%)					1.55	(1.18–2.02)	1.66	(1.27–2.18)

Final education	Junior high school/high school	1,324	177	(13.4%)					0.77	(0.57–1.04)	0.79	(0.59–1.07)
	Junior college/technical college	1,506	232	(15.4%)					0.88	(0.67–1.17)	0.93	(0.69–1.23)
	University	3,209	575	(17.9%)					0.97	(0.76–1.25)	0.98	(0.76–1.27)
	Graduate school	480	99	(20.6%)					ref.		ref.	

Number of people in household (including the respondent)	1	1,397	316	(22.6%)					ref.		ref.	
	≥2	5,122	767	(15.0%)					0.60	(0.52–0.70)	0.89	(0.83–0.95)

Marital status	Married	3,880	528	(13.6%)					0.76	(0.64–0.90)	0.81	(0.68–0.97)
	Not married	2,639	555	(21.0%)					ref.		ref.	

History of respiratory disease	Yes	987	209	(21.2%)							1.30	(1.09–1.56)
	No	5,532	874	(15.8%)							ref.	

History of mental illness	Yes	654	233	(35.6%)							2.93	(2.44–3.52)
	No	5,865	850	(14.5%)							ref.	

CI, confidence interval; COVID-19, coronavirus disease 2019; OR, odds ratio; Wfun, Work Functioning Impairment Scale.

Model 1: Adjusted for sex and age.

Model 2: Adjusted for sex, age, prefecture, use of combustible and heated tobacco products, occupational classification, working hours per week, final education, number of people in household, and marital status.

Model 3: Adjusted for sex, age, prefecture, use of combustible and heated tobacco products, occupational classification, working hours per week, final education, number of people in household, marital status, history of respiratory disease, and history of mental illness.

**Table 3.** Association between discomfort experienced due to odor and physiological responses of residual tobacco smoke brought into workplaces by smokers and mental health (K6)

			K6 score	Univariable analysis	Multivariable analysis							
	
			≥5		Model 1				Model 2		Model 3	
		*N*	*n*	(%)	OR	(95% CI)	OR	(95% CI)	OR	(95% CI)	OR	(95% CI)
Discomfort experienced due to odor of residual tobacco smoke brought into workplaces by smokers in the previous year	Never	5,407	2,044	(37.8%)	ref.		ref.		ref.		ref.	
	Sometimes	986	475	(48.2%)	1.53	(1.33–1.75)	1.50	(1.30–1.72)	1.50	(1.30–1.72)	1.46	(1.26–1.69)
	Frequently	126	64	(50.8%)	1.70	(1.19–2.42)	1.60	(1.12–2.29)	1.63	(1.13–2.34)	1.64	(1.13–2.37)

Sex	Male	3,380	1,266	(37.5%)			0.87	(0.79–0.96)	0.83	(0.74–0.93)	0.83	(0.74–0.94)
	Female	3,139	1,317	(42.0%)			ref.		ref.		ref.	

Age, years	20–39	2,045	1,010	(49.4%)			3.17	(2.72–3.69)	2.79	(2.35–3.31)	2.63	(2.21–3.14)
	40–59	3,086	1,252	(40.6%)			2.22	(1.92–2.56)	2.12	(1.81–2.47)	1.98	(1.69–2.32)
	≥60	1,388	321	(23.1%)			ref.		ref.		ref.	

Prefecture	High proportion of COVID-19 cases	3,638	1,433	(39.4%)					0.89	(0.80–0.99)	0.88	(0.79–0.98)
	Low proportion of COVID-19 cases	2,881	1,150	(39.9%)					ref.		ref.	

Use of combustible tobacco	Never smoked	4,569	1,869	(40.9%)					ref.		ref.	
	Past smokers	1,950	714	(36.6%)					1.08	(0.96–1.23)	1.01	(0.89–1.15)

Use of heated tobacco products	Never used	6,330	2,492	(39.4%)					ref.		ref.	
	Past users	189	91	(48.1%)					1.37	(1.00–1.87)	1.44	(1.05–1.97)

Occupational classification	Administrative and managerial workers/clerical workers	2,389	950	(39.8%)					ref.		ref.	
	Professional and technical workers	1,452	624	(43.0%)					1.05	(0.91–1.21)	1.06	(0.91–1.22)
	Sales workers/service workers/security workers	1,089	424	(38.9%)					0.89	(0.77–1.04)	0.89	(0.76–1.04)
	Others	1,589	585	(36.8%)					0.95	(0.83–1.10)	0.96	(0.83–1.11)

Working hours per week	<20	1,048	373	(35.6%)					ref.		ref.	
	20–29	786	267	(34.0%)					0.85	(0.70–1.04)	0.87	(0.71–1.07)
	30–39	1,337	523	(39.1%)					0.94	(0.79–1.13)	0.96	(0.80–1.16)
	40–49	2,525	1,039	(41.1%)					0.94	(0.80–1.11)	1.00	(0.84–1.18)
	≥50	823	381	(46.3%)					1.21	(0.99–1.49)	1.29	(1.04–1.59)

Final education	Junior high school/high school	1,324	485	(36.6%)					0.89	(0.71–1.13)	0.92	(0.73–1.17)
	Junior college/technical college	1,506	600	(39.8%)					0.95	(0.76–1.19)	1.00	(0.79–1.26)
	University	3,209	1,293	(40.3%)					0.99	(0.80–1.21)	1.01	(0.82–1.24)
	Graduate school	480	205	(42.7%)					ref.		ref.	

Number of people in household (including the respondent)	1	1,397	678	(48.5%)					ref.		ref.	
	≥2	5,122	1,905	(37.2%)					0.62	(0.52–0.74)	0.94	(0.89–0.99)

Marital status	Married	3,880	1,305	(33.6%)					0.67	(0.59–0.77)	0.71	(0.62–0.81)
	Not married	2,639	1,278	(48.4%)					ref.		ref.	

History of respiratory disease	Yes	987	473	(47.9%)							1.36	(1.17–1.57)
	No	5,532	2,110	(38.1%)							ref.	

History of mental illness	Yes	654	462	(70.6%)							3.78	(3.15–4.53)
	No	5,865	2,121	(36.2%)							ref.	

CI, confidence interval; COVID-19, coronavirus disease 2019; K6, Kessler Psychological Distress Scale; OR, odds ratio.

Model 1: Adjusted for sex and age.

Model 2: Adjusted for sex, age, prefecture, use of combustible tobacco and heated tobacco products, occupational classification, working hours per week, final education, number of people in household, and marital status.

Model 3: Adjusted for sex, age, prefecture, use of combustible tobacco and heated tobacco products, occupational classification, working hours per week, final education, number of people in household, marital status, history of respiratory disease, and history of mental illness.

## DISCUSSION

Using a nationwide Internet survey targeting non-smoking adult workers in Japan, the present study examined the discomfort experienced due to the odor and physiological responses of residual tobacco smoke brought into the workplace by smokers and its impact on work performance and mental health. Exposure to residual tobacco smoke is called thirdhand smoking; its components differ from those of secondhand smoking owing to chemical reactions, and there has been growing concern about its health effects in recent years.^2^^–^^4^ Although the experience of smelling residual tobacco smoke brought indoors by smokers is one of the components of exposure to thirdhand smoke, the actual situation and adverse effects have scarcely been investigated. Since it is not ethically feasible to conduct an RCT on such uncomfortable experiences, observational studies, such as ours, are essential to provide important scientific evidence. To the best of our knowledge, this is the first large-scale survey of such experiences of discomfort in a real-world setting.

The results of this study indicate that approximately 17.1% of non-smoking adult workers in Japan experienced discomfort due to residual tobacco smoke in their workplace at least once in the past year, with approximately 1.9% reporting frequent occurrences. Japan’s labor force in 2021 comprised 68.6 million people,^24^ of which 83.3% were non-smokers^25^ and 78.0% reported no exposure to passive smoking at work,^26^ it can be estimated that approximately 850,000 individuals frequently endured such discomfort while working. Smokers and companies that provide smoking areas must recognize the discomfort experienced by non-smokers due to residual tobacco smoke and attempt to reduce their discomfort, thereby contributing to the establishment of a comfortable work environment.

Our results show that the implementation of a smoke-free workplace policy is associated with a lower proportion of respondents experiencing discomfort. Previous reports on thirdhand smoke in indoor environments in domestic and public locations indicated that the degree of exposure to residual tobacco smoke is associated with the smoking rules of the location.^27^^–^^29^ Furthermore, it has been indicated that residual tobacco smoke accumulates over long periods, making non-smokers who move into homes previously inhabited by smokers more susceptible to the effects of thirdhand smoke.^30^ A study also reported that nicotine levels were the highest in hotel rooms where smoking was allowed, followed by non-smoking rooms in hotels with smoking rooms and hotels that were smoke-free.^31^ Therefore, enforcing complete no-smoking rules in the workplace is a viable solution for reducing the discomfort caused by the odor and physiological responses of residual tobacco smoke. Recently, some workplaces in Japan have implemented smoking policies, such as prohibiting the use of elevators for 45 minutes after smoking and banning entry to the premises.^32^ Other companies have implemented a no-smoking policy during work hours and a complete ban on smoking within the premises. Further advancements in such measures are necessary.

According to the analysis of the participants’ backgrounds, factors such as educational level and occupational classification were associated with the discomfort experienced due to the odor and physiological responses of residual tobacco smoke in the workplace. Individuals with lower levels of education were more likely to report such experiences, which is consistent with the findings of a survey on thirdhand smoke exposure in South Korea.^33^ In terms of occupational classification, individuals employed in sales and marketing (eg, sales, retail, real estate, insurance sales), service industries (eg, beauticians, waiters, home helpers), and security positions (eg, self-defense officials, police, firefighters) were more likely to have such experiences. This suggests that occupations that involve frequent contact with a diverse range of people in various environments are more likely to be exposed to residual tobacco smoke.

An important finding of this study is that individuals who frequently experience discomfort due to the odor and physiological responses of residual tobacco smoke tend to have higher WFun and K6 scores. This association was consistent even after adjusting for potential confounding factors and stratification by a history of respiratory diseases or mental illness. This may be attributed to the psychological stress response induced by exposure to the unpleasant odor of residual tobacco smoke, in addition to physiological responses caused by hazardous chemical compounds. Unpleasant odors activate the brain’s limbic system, which is directly connected to the sense of smell and emotional responses. This activation can immediately affect emotions and mood, leading to increased stress.^34^ Stress is widely recognized for its adverse effects on physical and psychological health.^35^ In the workplace environment, stress was reported as a significant cause of presenteeism and mental health issues.^36^ In particular, with an increase in presenteeism, the discomfort from residual tobacco smoke may deteriorate the work environment. It was suggested that poor work environments are directly associated with reduced work efficiency.^37^ Furthermore, unpleasant environmental factors can impair concentration and cognitive efficiency, potentially contributing to increased presenteeism and reduced work capacity.^38^ Therefore, from an occupational health perspective, enforcing stricter smoking regulations in the workplace and implementing measures to protect employee health are urgently required.

### Limitations

This study had certain limitations. First, as this is a cross-sectional study, it is limited in establishing causation. A future longitudinal approach could strengthen our findings by tracking changes in discomfort, work performance, and mental health over time, providing insight into how repeated or prolonged exposure may impact employees. Such an approach could also help clarify both immediate and long-term effects of discomfort in workplace settings, supporting more robust policy recommendations. Second, the JASTIS survey was conducted with a convenience sample of those who voluntarily registered as Internet research panelists. Therefore, the study participants may not be representative of all non-smoking adult workers in Japan. Third, as the study was based on an Internet survey, there is a possibility that some participants responded without thinking to minimize the effort involved in completing the survey (ie, satisficing). Although we conducted a response consistency check and excluded invalid responses from the analyses, the data quality may have been affected. Fourth, considering that the regulations of tobacco products and other environmental conditions may vary by country, the results of our study may not apply to other countries. Thus, these limitations may cause an overestimation or underestimation of the outcomes of interest, and our results should be carefully interpreted in terms of limited generalizability, reliability, and validity.

### Conclusions

Using data from the JASTIS 2021 survey, this study revealed the discomfort experienced due to the odor and physiological responses of residual tobacco smoke brought into the workplace by smokers, targeting adult workers without firsthand or secondhand smoking in Japan. The frequency of such experiences was independently associated with poor work performance and mental health. Efforts to promote strict smoke-free workplace policies (such as prohibiting smoking in all areas) are a possible solution for reducing such experiences.

## SUPPLEMENTARY MATERIAL

eTable 1. Association between discomfort experienced due to odor and physiological responses of residual tobacco smoke brought into workplaces by smokers and presenteeism (WFun) according to medical history of respiratory disease/mental illness

eTable 2. Association between discomfort experienced due to odor and physiological responses of residual tobacco smoke brought into workplaces by smokers and mental health (K6) according to medical history of respiratory disease/mental illness
