# Supplementary material for: Discomfort Experienced Due to the Odor and Physiological Responses of Residual Tobacco Smoke Brought Into Workplaces by Smokers on Work Performance and Mental Health
Source: J Epidemiol. 2025 Sep 5;35(9):393–401. doi: 10.2188/jea.JE20240354 (PMC12358253; doi:10.2188/jea.JE20240354)
Supplement: Supplementary file 1 [file je-35-393-s001.pdf]

eTable 1. Association between discomfort experienced due to odor and physiological responses of residual tobacco smoke brought into workplaces by smokers and presenteeism (WFun) according to medical history of respiratory disease/mental illness

Medical history	Discomfort experienced in the previous year		WFun score ≥21		Univariate analysis		Multivariable analysis Model 1		Model 2		Model 3	
		N	*n*	(%)	OR	(95% CI)	OR	(95% CI)	OR	(95% CI)	OR	(95% CI)
With respiratory disease	Never	786	155	(19.7%)	ref.		ref.		ref.		ref.	
	Sometimes	185	50	(27.0%)	1.51	(1.04–2.18)	1.41	(0.97–2.05)	1.39	(0.95–2.05)	1.42	(0.96–2.09)
	Frequently	16	4	(25.0%)	1.36	(0.43–4.27)	1.18	(0.37–3.75)	1.31	(0.4–4.28)	1.10	(0.33–3.68)
Without respiratory disease	Never	4,621	685	(14.8%)	ref.		ref.		ref.		ref.	
	Sometimes	801	160	(20.0%)	1.43	(1.19–1.74)	1.41	(1.16–1.71)	1.42	(1.17–1.73)	1.38	(1.13–1.68)
	Frequently	110	29	(26.4%)	2.06	(1.34–3.17)	1.93	(1.25–2.98)	1.97	(1.26–3.06)	2.00	(1.28–3.14)
With mental illness	Never	519	173	(33.3%)	ref.		ref.		ref.		ref.	
	Sometimes	121	53	(43.8%)	1.56	(1.04–2.33)	1.57	(1.04–2.36)	1.59	(1.04–2.43)	1.59	(1.04–2.43)
	Frequently	14	7	(50.0%)	2.00	(0.69–5.79)	1.79	(0.61–5.23)	2.04	(0.68–6.16)	2.02	(0.67–6.1)
Without mental illness	Never	4,888	667	(13.6%)	ref.		ref.	(0.61–5.23)	ref.		ref.	
	Sometimes	865	157	(18.2%)	1.40	(1.16–1.7)	1.36	(1.12–1.65)	1.39	(1.14–1.69)	1.37	(1.12–1.66)
	Frequently	112	26	(23.2%)	1.91	(1.23–2.99)	1.79	(1.14–2.81)	1.79	(1.13–2.82)	1.80	(1.14–2.85)

Wfun, Work Functioning Impairment Scale.
Model 1: Adjusted for sex and age.

Model 2: Adjusted for sex, age, prefecture, use of combustible tobacco and heated tobacco products, occupational classification, working hours per week, final education level, number of people in the household, and marital status.

Model 3: Adjusted for sex, age, prefecture, use of combustible tobacco and heated tobacco products, occupational classification, working hours per week, final education level, number of people in the household, marital status, and history of respiratory disease/history of mental illness.

eTable 2. Association between discomfort experienced due to odor and physiological responses of residual tobacco smoke brought into workplaces by smokers and mental health (K6) according to medical history of respiratory disease/mental illness

Medical history	Discomfort experienced in the previous year		K6 score ≥5		Univariate analysis		Multivariable analysis Model 1		Model 2		Model 3	
		N	*n*	(%)	OR	(95% CI)	OR	(95% CI)	OR	(95% CI)	OR	(95% CI)
With respiratory disease	Never	786	350	(44.5%)	ref.		ref.		ref.		ref.	
	Sometimes	185	113	(61.1%)	1.96	(1.41–2.71)	1.89	(1.36–2.63)	1.93	(1.37–2.72)	1.99	(1.41–2.82)
	Frequently	16	10	(62.5%)	2.08	(0.75–5.77)	1.95	(0.7–5.47)	2.09	(0.74–5.92)	1.79	(0.62–5.19)
Without respiratory disease	Never	4,621	1694	(36.7%)	ref.		ref.		ref.		ref.	
	Sometimes	801	362	(45.2%)	1.43	(1.23–1.66)	1.40	(1.2–1.64)	1.40	(1.2–1.64)	1.38	(1.17–1.62)
	Frequently	110	54	(49.1%)	1.67	(1.14–2.43)	1.57	(1.07–2.31)	1.59	(1.07–2.34)	1.61	(1.09–2.39)
With mental illness	Never	519	355	(68.4%)	ref.		ref.		ref.		ref.	
	Sometimes	121	94	(77.7%)	1.61	(1.01–2.56)	1.62	(1.01–2.62)	1.56	(0.95–2.56)	1.56	(0.95–2.56)
	Frequently	14	13	(92.9%)	6.01	(0.78–46.30)	5.24	(0.68–40.56)	5.90	(0.75–46.29)	5.83	(0.74–45.78)
Without mental illness	Never	4,888	1689	(34.6%)	ref.		ref.		ref.		ref.	
	Sometimes	865	381	(44.0%)	1.49	(1.29–1.73)	1.46	(1.26–1.7)	1.47	(1.27–1.71)	1.45	(1.25–1.69)
	Frequently	112	51	(45.5%)	1.58	(1.09–2.31)	1.50	(1.02–2.2)	1.49	(1.01–2.19)	1.51	(1.02–2.22)

K6, Kessler Psychological Distress Scale.

Model 1: Adjusted for sex and age.

Model 2: Adjusted for sex, age, prefecture, use of combustible tobacco and heated tobacco products, occupational classification, working hours per week, final education level, number of people in the household, and marital status.

Model 3: Adjusted for sex, age, prefecture, use of combustible tobacco and heated tobacco products, occupational classification, working hours per week, final education level, number of people in the household, marital status, and history of respiratory disease/history of mental illness.
